# Study on the Dose–Response Relationship between Magnesium and Type 2 Diabetes of Childbearing Women in the China Adult Chronic Disease and Nutrition Surveillance 2015

**DOI:** 10.3390/nu16071018

**Published:** 2024-03-31

**Authors:** Huidi Zhang, Jingxin Yang, Yang Cao, Xiaoyun Shan, Lichen Yang

**Affiliations:** Key Laboratory of Public Health and Nutrition, National Health Commission of the People’s Republic of China, National Institute for Nutrition and Health, Chinese Center for Disease Control and Prevention, Beijing 100050, China; zhanghuidi1114@126.com (H.Z.); jingxinyang2022@126.com (J.Y.); yasmine0814@163.com (Y.C.); shanxiaoyun0924@163.com (X.S.)

**Keywords:** magnesium, dose–response relationship, threshold value, type 2 diabetes, childbearing women

## Abstract

**Background**: Magnesium (Mg) is an essential element and participates in many metabolic pathways. Many studies have found a certain negative correlation between magnesium and blood glucose parameters, but the dose–response relationship between them is still a relatively narrow research field. We aim to explore the dose–response relationship between plasma and dietary Mg and type 2 diabetes (T2DM) among childbearing women in a nationally representative sample. And we will also initially explore the threshold of dietary and plasma magnesium in the prevention of T2DM and their consistency. **Methods**: A total of 2912 18–44 year-old childbearing women were recruited from the China Adult Chronic Disease and Nutrition Surveillance (2015). Multivariate logistic regression was used to explore the dose–response relationship between plasma and dietary Mg and glucose parameters. The threshold effect between Mg and T2DM was explored by a restricted cubic spline regression. **Results**: It was found that when plasma Mg was increased by 0.041 mmol/L, the risk of T2DM, impaired fasting glucose (IFG), and HbA1c-hyperglycemia was reduced by 18%, 19%, and 18%, respectively. The possible threshold value for plasma Mg to prevent the risk of T2DM was 0.87 mmol/L. Through the quality control of the sample dietary survey data, 2469 cases were finally included for dietary analysis. And the possible threshold value for dietary Mg to prevent the risk of T2DM was 408 mg/d. Taking the recommended dietary Mg intake of 330 mg/d as the reference group, when the Mg intake reached 408 mg/d, the risk of T2DM was significantly reduced. And the average plasma Mg level of the people whose dietary intake reached 408 mg/d was 0.87 mmol/L. **Conclusions**: These results indicate that dietary Mg and plasma Mg have good consistency on the threshold effect of glucose parameters in women of childbearing age.

## 1. Introduction

Magnesium is an essential element in humans [1]. As a cofactor in more than 300 enzymatic reactions, magnesium plays an important physiological role in many functions of the human body, including protein synthesis, blood sugar control, and blood pressure regulation [2]. Based on its multiple functions, a low magnesium level is associated with the occurrence and development of many chronic diseases, such as Alzheimer’s disease, insulin resistance, T2DM, and hypertension [3].

The association between hypomagnesemia and T2DM has aroused the great interest of researchers since it was first discovered by Beckett and Lewis [4] in 1959. There is evidence that the relationship between magnesium deficiency and T2DM is bidirectional. Magnesium deficiency is a common manifestation in patients with type 1 and T2DM [5,6], and magnesium deficiency may also increase the risk of diabetes [7,8].

Many studies [9,10,11] show that with the increase of blood magnesium level, the risk of T2DM is significantly reduced. And epidemiological and multicenter studies have also shown an inverse relationship between the intake of magnesium-rich foods and the risk of diabetes [12]. Although some studies have suggested that there may be a threshold between plasma magnesium and T2DM, there are very limited research reports on the specific dose–response relationship and threshold effect between magnesium and T2DM.

In this study, we intend to analyze the dose–response relationship between either dietary or plasma magnesium and T2DM through nationally representative population data. At the same time, we will preliminarily explore the consistency of the threshold effect of dietary and plasma magnesium on glucose-related parameters in Chinese childbearing women.

## 2. Materials and Methods

### 2.1. Subjects

The data for the study came from the 2015 Chinese Adult Chronic Disease and Nutrition Surveillance, a nationally representative cross-sectional survey. A stratified, multi-stage, probabilistic random selection approach was used to pick all survey participants. The sampling method was described in detail in Yu et al. [13]. Considering regional types and monitoring sites, participants of reproductive age were chosen at random from the overall population for this investigation. Samples with incomplete questionnaire responses, poor blood quality (such as hemolysis), and biological index values below the detection limit were also disregarded. This study population did not include pregnant women and nursing mothers, and this particular population was monitored in a separate study. This survey eventually comprised 2912 childbearing women. Each participant gave their written consent after being fully informed. The protocol was approved by the Ethics Committee of the National Institute of Nutrition and Health, Chinese Center for Disease Control and Prevention (No: 201519-A), and the study was carried out in line with the Declaration of Helsinki.

### 2.2. Basic Information and Sample Collection

Basic demographic data were gathered by consistently trained investigators using a standardized questionnaire. Nationality, education, and drinking habits were collected using a self-reported questionnaire. There were three categories for education levels: elementary (primary school and lower), medium (junior high/high school/secondary school), and advanced (junior college or above). The eastern, middle, and western parts of the district were separated. Anthropometric measures were also taken by skilled medical personnel using defined protocols. Measurements of blood pressure, waist circumference, height, and weight are part of the physical examination. Height and weight were used to determine the body mass index (BMI).

After at least 10 h of fasting, venous blood was drawn in the morning. Each blood sample was separated into an anticoagulant tube and a serum separator tube. Between 20–30 min after the blood was drawn, plasma separator tubes containing blood samples were immediately centrifuged at 3000× *g* for 15 min. Afterward, the plasma aliquots were separated and stored at 80 °C for further testing.

### 2.3. Plasma Mg and Laboratory Index Detection

An automated biochemical analyzer (Hitachi 7600, Tokyo, Japan) was used to evaluate the concentrations of serum fasting glucose, high density lipoprotein cholesterol (HDL-C), low density lipoprotein cholesterol (LDL-C), total cholesterol (TC), and triglyceride (TG). And glycated hemoglobin (HbA1c) was measured using HPLC (Waters E2695, Milford, MA, USA).

Inductively coupled plasma mass spectrometry (ICP-MS, PerkinElmer, NexION 350, Waltham, MA, USA) was used to detect the amounts of magnesium and calcium. The plasma element was measured by 0.5% (*v*/*v*) high-purity nitric acid dilution (1:20). The precision and accuracy of the analysis were checked at 10-sample intervals using the quality control samples (Seronorm, Level-2, Billingstad, Norway).

The coefficient of variation for Mg was 2.33% for between batches and 1.19% for within batches. The coefficient of variation for Ca was 1.23% for between batches and 2.62% for within batches. The recovery of Mg and Ca were 100.10% and 97.63%, respectively.

### 2.4. Evaluation Standards of Glucose Parameters

T2DM, impaired fasting glucose, and HbA1c hyperglycemia all satisfied the respective diagnostic standards advised by the World Health Organization in 2006 [14]. Fasting glucose ≥ 7.0 mmol/Land/or 2 h post-glucose load ≥ 11.1 mmol/L and/or HbA1c ≥ 6.5% were used to define T2DM. Fasting glucose between 6.1 and 7.0 mmol/L was referred to as impaired glucose tolerance (IFG). HbA1c levels above 6.5% are considered to be HbA1c-hyperglycemia.

### 2.5. Calculation and Quality Control of Dietary Magnesium

A validated food frequency questionnaire (FFQ) was used in CNHS 2015 to gather data on participants’ dietary habits during the previous 12 months [15]. The questionnaire contained 64 items on it. The edible weight was defined as the weight ingested in a single day, and the daily eating weight was determined depending on frequency (daily, weekly, monthly, or yearly). The China Food Composition Table (2018) [16] was used to determine each person’s daily intake of dietary energy, macronutrients, calcium (Ca) (mg), and magnesium (Mg). Since magnesium supplements are not common in China, this study did not take their usage into account. From the 2912 cases, the samples with complete dietary data and energy intake between 800–4000 kcal meeting the quality control requirements were selected. Finally, 2469 samples were included for follow-up analysis of dietary data. The residuals model was also used to modify dietary magnesium intake for total calorie consumption [17].

### 2.6. Data Analyses

Statistical analyses were performed using SPSS version 19.0, SAS 9.3, and R 4.0.3. The possible influencing factors of Mg were analyzed by a generalized additive model. After adjusting the confounding factors affecting blood glucose parameters, the dose–response relationship between blood glucose parameters and plasma magnesium concentration was evaluated by a multiple logistic regression model, which could be described as the odds ratios (ORs) and 95% confidence intervals (95% CIs). And the multiple logistic regression was also used to calculate the relationship between dietary Mg and blood glucose parameters. The threshold effect between plasma and dietary magnesium and blood glucose parameters was explored by a restricted cubic spline regression. All statistical tests were two-sided, and statistical significance was considered at *p* < 0.05.

## 3. Results

### 3.1. Plasma Magnesium Distribution of 2912 Childbearing Women

This study was conducted on 2912 childbearing women, after excluding the hemolysis, incomplete data, and other unqualified samples. Table 1 describes plasma magnesium concentration levels and deficiency rates under total and different blood glucose conditions. At the same time, the magnesium level and deficiency rate under different group variables are also described. The median of total magnesium in 2912 subjects was 0.86 mmol/L, and the magnesium deficiency rate was 7.73% (when the plasma magnesium level equal to or less than 0.75 mmol/L is considered as a deficiency).

The median values of magnesium in the NFG, IFG, and T2DM group were 0.87 mmol/L, 0.85 mmol/L, and 0.84 mmol/L respectively, indicating a downward trend; the deficiency rate of magnesium was 4.30%, 9.94%, and 16.70%, respectively, showing an upward trend.

Under different stratification factors, the distribution of plasma magnesium concentration in the NFG, IFG, and T2DM group, except for the 18–25 age group, showed a downward trend. The distribution of the magnesium deficiency rate in the NFG, IFG, and T2DM group, except for the underweight factor of BMI, all showed an upward trend, and the magnesium deficiency rate of the three groups had statistical significance in each level.

### 3.2. The Basic Characteristic of 2912 Subjects

Table 2 shows the clinical characteristics of 2912 Chinese women of childbearing age. The comparison among the three groups showed that height, weight, waist circumference, SBP, DBP, TG, TC, LDL-C, and UA increased with the increase of the glucose parameters levels, while HDL decreases as glucose parameters rise. The differences among all groups were statistically significant (*p* < 0.01).

### 3.3. The Associations of Plasma Magnesium Concentration with Glucose Parameters

Table 3 shows the odds ratios for T2DM, IFG, and HbA1c hyperglycemia in relation to the levels of plasma magnesium concentrations as continuous variables and is divided into quintiles based on the distribution. Lower plasma Mg concentrations were linked with higher ORs for T2DM, IFG, and HbA1c-hyperglycemia. For T2DM, IFG, and HbA1c-hyperglycemia, a magnesium level less than 0.75 mmol/L is a risk factor (all *p* values were less than 0.01); the odds ratios were 3.99, 4.05, and 3.90, respectively. And adjusting for additional potential confounders did not significantly alter the results. Additionally, Mg levels between 0.75 and 0.85 mmol/L are associated with an increased risk of T2DM, IFG, and HbA1c hyperglycemia (all *p* values were less than 0.05); the odds ratios were 1.55, 1.49, and 1.68, respectively. The results were almost unchanged after accounting for potential confounders. When plasma magnesium was used as an ordered variable, there was a tendency to decrease the risk of T2DM, IFG, and HbA1c hyperglycemia with increasing magnesium levels.

In the dose–response relationship, plasma magnesium was used as a continuous variable to explore the relationship between serum magnesium and abnormal blood glucose. It was found that with every 0.041 mmol/L increase in the plasma magnesium level, the risk of T2DM, IFG, and HbA1c-hyperglucose was reduced compared with people with normal blood glucose, and the OR values were 0.82, 0.81, and 0.82, respectively. In other words, it means when plasma magnesium was increased by 0.041 mmol/L, the risk of T2DM, IFG, and HbA1c-hyperglycemia was reduced to 18%, 19%, and 18%, respectively.

### 3.4. The Dose–Response Relationship between Plasma Mg and the Risk of T2DM

**Figure 1:**
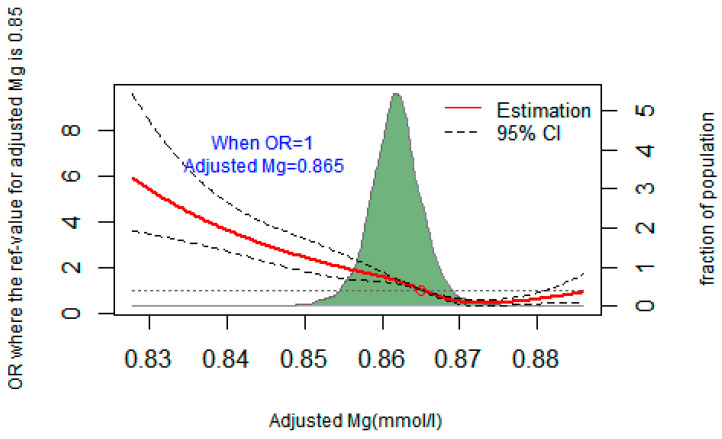
Restricted cubic spline regression fitting curve of adjusted plasma magnesium and T2DM.

A restricted cubic spline regression was used to fit the relationship between adjusted plasma magnesium and T2DM, as shown in Figure 1. When the OR value of T2DM risk was 1, the corresponding adjusted plasma magnesium concentration was 0.865 mmol/L. The risk of T2DM increased when plasma magnesium was less than 0.865 mmol/L (OR > 1). Lines represent ORs (solid line) and 95% CIs (dashed line) based on restricted cubic splines for plasma magnesium concentrations. Confounding factors such as age, sex, BMI, drink, systolic and diastolic blood pressure, and plasma calcium were adjusted for plasma magnesium. The points where the lines coincide represent the adjusted plasma magnesium concentration when the OR value is equal to 1.

### 3.5. Daily Dietary Nutrient Intake of the Subjects

The daily dietary nutrient intake of 2469 subjects after quality screening are shown in Table 4. There was no statistical difference in nutrient intake among the three groups.

### 3.6. The Associations of Dietary Magnesium Concentration with Glucose Parameters

Table 5 shows the multivariate logistic analysis of the correlations between dietary Mg intake and glucose parameters. With a dietary magnesium intake of less than 321.35 mg/d as a reference, when the Mg intake reached 439.47 mg/d, the risk of IFG was reduced. The results were consistent after adjusting the confounders. During the analysis of T2DM, it was also found that when taken, with a Mg intake of less than 321.35 mg/d as a reference, the Mg intake between 439.47–<492 mg/d would reduce the risk of T2DM.

### 3.7. The Dose–Response Relationship between Dietary Mg and the Risk of T2DM

**Figure 2:**
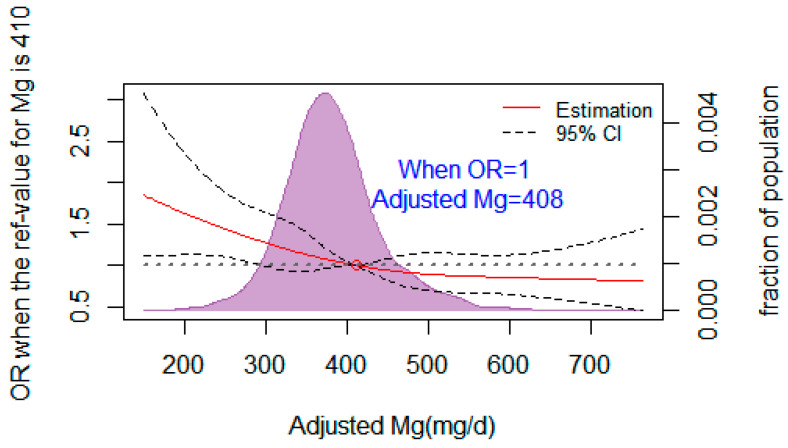
Restricted cubic spline regression fitting curve of adjusted dietary magnesium and T2DM.

Figure 2 illustrates the dose–response relationship between dietary Mg and T2DM using a restricted cubic spline regression. The corresponding adjusted dietary Mg was 408 mg/d when the OR value of the risk of T2DM was 1. The lines represent ORs (solid line) and 95% CIs (dashed line) based on restricted cubic splines. Dietary Mg was corrected for confounding variables such as age, sex, BMI, drink, systolic and diastolic blood pressure, and dietary calcium. The points where the lines coincide represent the adjusted dietary Mg concentration when the OR value is equal to 1.

### 3.8. Consistency Analysis of Dose–Response Effect of Dietary Magnesium and Plasma Magnesium on Glucose Parameters

Table 6 shows the consistency analysis of threshold effects of dietary magnesium and plasma magnesium on glucose parameters. Taking the recommended dietary Mg intake of 330 mg/d as the reference group, when the Mg intake reached 408 mg/d, the risk of IFG and T2DM were both significantly reduced. And the average plasma magnesium level of the people whose dietary intake reached 408 mg/d was 0.87 mmol/L. These results indicate that dietary Mg and plasma Mg have good consistency on the threshold effect of glucose parameters in women of childbearing age.

## 4. Discussion

Since the 1940s, studies have shown that T2DM was associated with hypomagnesemia [18]. A low serum magnesium level was found in a large number of T2DM cohort studies [19]. Magnesium deficiency affects the blood glucose regulation and metabolic function of diabetes patients, and diabetes and insulin resistance can further deepen magnesium deficiency [8]. Therefore, fully understanding and exploring the dose–response relationship between magnesium and T2DM will not only help to further explore practical T2DM intervention measures to provide an important basis, but also provide scientific data for the prevention and treatment of hypomagnesemia in the future.

In this study, we first studied the dose–response relationship between plasma magnesium and blood glucose parameters. The distribution of plasma magnesium was divided according to five quantiles, and 0.85–0.95 was used as a reference. It was found that the risk of T2DM, IFG, and HbA1c-hyperglucose were significantly increased when the plasma Mg level was less than 0.85 mmol/L.

The results are similar to those reported in several countries. In a cross-sectional study of adults in Korea [20], the risk of hyperglycemia was significantly increased when plasma magnesium levels were lower than 0.80 mmol/L as a reference (OR = 2.28, 95% confidence interval 1.29–4.02). In the Australian population [21], when serum magnesium was lower than 0.79 mmol/L as a reference, the risk of T2DM and IFG increased to 393% and 145%, respectively. In a 10-year follow-up study of the Mexican population [22], when serum magnesium was lower than 0.74 mmol/L, the risk of T2DM and IFG increased by 254% and 149%, respectively. In a study of a middle-aged and elderly Chinese population [23], it was found that when serum magnesium was higher than 0.87 mmol/L, the risk of developing T2DM was reduced by 0.34 times. (OR = 0.34, 95% CI 0.24–0.49). Although different studies have different blood magnesium threshold values for evaluating the risk of T2DM, they all suggest a dose–effect relationship between them.

In this study, when plasma magnesium was analyzed as a continuous variable, it was found that the risk of T2DM, IFG, and HbA1c-hyperglucose was reduced by 18%, 19%, and 18% for each 0.041 mmol/L increase in plasma Mg, respectively. Data from the Nutrition and Health Monitoring of Canadian Residents [24] show that compared with people with normal blood glucose, a decrease in the serum magnesium level of 0.04–0.07 mmol/L increases the risk of T2DM. A cohort study [25] of 1999 Japanese patients followed for 15.6 years showed that for every standard deviation increase in Mg levels, the risk of T2DM was 14% lower (*p* = 0.04). Another study in Wuhan, Hubei Province [26], also found that the risk of T2DM and IFG decreased by 10% and 24%, respectively, for each 0.041 mmol/L increase in plasma Mg. The inconsistencies in the value of the dose–response relationship between plasma Mg and glucose parameters may be attributed to differences in the study population, the measurement of magnesium, and the prevalence of diabetes.

Many studies [27,28] believed that the lower limit of reference range for plasma Mg in T2DM patients should be 0.85 mmol/L. In a cohort of 9784 subjects followed by NHANES I for 18 years [29], it was found that the risk ratio for T2DM began to increase when Mg was less than 0.85 mmol/L. The OR value for the risk of T2DM was 1.20 when Mg was between 0.80 and 0.84 mmol/L, and 1.51 when Mg was <0.80 mmol/L. Recent studies have shown [30,31] that individuals with plasma Mg less than 0.75 mmol/L are most likely to suffer from Mg deficiency, while a plasma Mg level above 0.85 mmol/L may be the lower limit of a suitable level to maintain human health. However, the above studies are observational results based on large sample populations, rather than statistical methods to explore statistically significant thresholds. In a case-control study on serum Mg and hyperglycemia [26], the possible threshold of plasma Mg in preventing the occurrence of T2DM was explored by a restricted cubic spline regression. After adjusting for potential confounders of Mg, it was found that when Mg was lower than 0.91 mmol/L, the risk of prediabetes and T2DM was significantly reduced, and then no significant decline was observed. In this study, we found that the threshold value for plasma Mg to prevent T2DM was 0.87 mmol/L, which is similar to the threshold recommended by the above research.

Studies have also shown that there is a significant dose–response relationship between dietary Mg intake and T2DM [24,32]. Whether in American black women [33] with 115 mg/d Mg intake, in Japanese people [34] with less than 300 mg/d Mg intake, or in German people [35] with less than 350 mg/d Mg intake, dietary Mg intake is negatively correlated with the risk of T2DM. In this study, through the analysis of the FFQ among 2469 subjects, we found that the total dietary energy intake of women of childbearing age was 1964 kcal. The intake of the protein, fat, and carbohydrate were all within the normal range. By comparing the sextile, it was found that the higher the dietary magnesium intake, the lower the risk of IFG and T2DM when compared with the recommended dietary magnesium intake of Chinese people (320 mg/d). And the results were consistent with Song Y et al. [36] and Hruby et al. [37]. We also explored the possible threshold of dietary magnesium in the prevention of T2DM by a restricted cubic spline regression. It was found that there was a significant association with T2DM when the diet Mg intake was less than 408 mg/d [38]. This threshold is very close to our team’s previously published research, with a threshold of 410 mg/d for dietary magnesium in the prevention of prediabetes. However, this study is different from the results of the limited studies on the threshold effect. A meta-analysis indicated that 300 mg/day of Mg intake was the essential dose for preventing T2DM [39]. A cohort study showed that the risk of metabolic syndrome (MetS) and its components decreased significantly with the increase of magnesium intake when the magnesium intake was less than 280 mg/day [40]. However, a cross-sectional study concluded that more than 300 mg/d of Mg intake might not improve insulin sensitivity and have no influence [41]. The difference in the dietary magnesium threshold may be due to different dietary survey methods (including 3 days, 24 h, and FFQ) [38] and statistical methods, and another may be due to differences among study populations. However, unlike other studies, we further analyzed the threshold effects of dietary and plasma Mg on T2DM and found a good consistency between plasma and dietary Mg.

This study is the first to evaluate and analyze the dose–response relationship between Mg nutritional status and T2DM in Chinese women of childbearing age based on nationally representative surveillance. In this study, we also preliminarily proposed thresholds for Mg to reduce the risk of T2DM. At the same time, we found that dietary Mg and plasma Mg had a good consistency on the threshold effect of blood glucose in women of childbearing age. Many epidemiological studies have found a high prevalence of hypomagnesemia in patients with type 2 diabetes mellitus (T2DM), especially in those with poor glycemic control [7,42]. At present, a large number of literature reports show that magnesium deficiency will bring a series of adverse effects on the body and even accelerate the occurrence and development of many chronic diseases. Therefore, timely and effective assessment of magnesium nutrition status of the population is of great significance for the prevention of chronic diseases. At present, there is no systematic assessment of the magnesium nutrition status of the population based on nationally representative data in China. Randomized controlled trials (RCTS) are key to establishing and validating systems for external exposure to influence internal exposure levels and thereby improve health outcomes. Although randomized controlled trials of magnesium supplementation have been reported in the literature, there is a wide variation between studies and inconsistent results. However, there have been no randomized controlled trials of magnesium supplements in China. At present, our team has completed a study on the improvement of the blood glucose regulation effect of dietary magnesium supplementation in elderly T2DM patients with hypomagnesemia, and the study results are under review. It is believed that the causal relationship between hypomagnesemia and T2DM can be explored in the future through the cross-sectional studies, intervention studies, and future mechanism studies completed by our team.

The study also has some limitations. The first limitation of this study lies in the type of cross-sectional study, which can only observe the correlation between variables, rather than the accidental relationship between variables. And the second one is the narrow age range and population of this study make it unclear whether the relationship can be replicated in other populations.

## 5. Conclusions

In summary, according to the latest monitoring results in 2015, the average plasma level of magnesium in 2912 childbearing women in China is 0.86 mmol/L. It was found that when plasma Mg was increased by 0.041 mmol/L, the risk of T2DM, IFG, and HbA1c-hyperglycemia was reduced 18%, 19%, and 18%, respectively. The possible threshold value for plasma Mg to prevent the risk of T2DM was 0.87 mmol/L. And the possible threshold value for dietary Mg to prevent the risk of T2DM was 408 mg/d. With the recommended dietary magnesium intake of 330 mg/d as the reference group, the risk of T2DM decreased when the magnesium intake reached 408 mg/d and the corresponding average plasma magnesium level was 0.87 mmol/L. Dietary Mg and plasma Mg have good consistency on the threshold effect of glucose parameters in women of childbearing age. Further studies are needed to confirm our findings in more populations and clarify the mechanisms behind this relationship.

## Figures and Tables

**Table 1:** The distribution and comparison of Mg concentrations among different groups in 2912 subjects (mmol/L).

Characteristics	Total				NFG				IFG				T2DM				*p* *
	N	M ^#^	P25–P75	D * (%)	N	M ^#^	P25–P75	D * (%)	N	M ^#^	P25–P75	D * (%)	N	M ^#^	P25–P75	D * (%)	
Total	2912	0.86	7.73	7.73	1811	0.87	0.09	4.31	529	0.86	0.11	9.83	572	0.84	0.11	16.61	0.001
Age group																	
18–25 y	717	0.86	0.10	7.53	638	0.87	0.1	5.96	30	0.86	0.14	13.33	49	0.86	0.18	24.49	0.001
26–35 y	932	0.86	0.09	7.19	619	0.88	0.09	3.55	160	0.85	0.10	10.00	153	0.83	0.11	18.95	0.001
36–45 y	1263	0.86	0.10	8.23	554	0.88	0.1	3.25	339	0.86	0.11	9.44	370	0.84	0.11	14.59	0.001
District																	
Eastern	972	0.86	0.10	8.02	590	0.88	0.11	3.22	180	0.86	0.09	8.89	202	0.83	0.11	21.29	0.001
Central	998	0.86	0.10	7.92	647	0.87	0.09	5.56	153	0.86	0.11	9.80	198	0.83	0.11	14.14	0.001
Western	942	0.87	0.10	7.22	574	0.88	0.09	4.01	196	0.85	0.11	10.71	172	0.85	0.12	13.95	0.001
City type																	
City	1359	0.86	0.10	7.73	736	0.87	0.10	3.26	304	0.86	0.09	7.57	319	0.83	0.11	18.18	0.001
Rural	1553	0.86	0.10	7.73	1075	0.87	0.09	5.02	225	0.85	0.11	12.89	253	0.84	0.11	14.62	0.001
Nationality																	
Han	2548	0.86	0.10	7.54	1594	0.87	0.10	4.39	459	0.86	0.10	9.80	495	0.84	0.11	15.56	0.001
Ethnic minorities	364	0.86	0.10	9.07	217	0.87	0.08	3.69	70	0.86	0.12	10.00	77	0.85	0.14	23.38	0.001
BMI																	
Underweight	174	0.88	0.09	7.47	144	0.88	0.09	4.86	10	0.81	0.17	30.00	20	0.84	0.15	15.00	0.001
Normal	1415	0.87	0.10	6.78	1027	0.88	0.10	3.99	195	0.86	0.11	8.72	193	0.84	0.12	19.69	0.001
Overweight	856	0.86	0.09	7.83	445	0.87	0.09	4.94	200	0.86	0.09	8.00	211	0.84	0.10	13.74	0.001
Obesity	467	0.85	0.11	10.49	195	0.86	0.10	4.10	124	0.85	0.11	12.90	148	0.83	0.12	16.89	0.001
Education																	
Primary	923	0.86	0.10	8.78	451	0.88	0.09	4.43	234	0.85	0.11	9.83	238	0.83	0.12	15.97	0.001
Medium	1564	0.86	0.10	7.74	1047	0.87	0.09	4.39	250	0.85	0.10	10.80	267	0.84	0.11	17.98	0.001
Advanced	425	0.87	0.10	5.41	313	0.88	0.09	3.83	45	0.87	0.10	4.44	67	0.85	0.11	13.43	0.019
Drink																	
Yes	596	0.86	0.10	6.71	385	0.87	0.09	4.16	103	0.86	0.09	6.80	108	0.85	0.11	15.74	0.001
No	2316	0.86	0.10	7.99	1426	0.88	0.10	4.35	426	0.85	0.11	10.56	464	0.84	0.11	16.81	0.001

M ^#^: median value; D *: plasma Mg deficiency; NFG refers to normal fasting glucose; *p* *, *p* value for deficiency; P25–P75: indicates the 25th to 75th quantile.

**Table 2:** Basic characteristics of the subjects.

Index	Total (*n* = 2469)	NFG (*n* = 1533)	IFG (*n* = 462)	T2DM (*n* = 451)	*p* Value
Height (cm)	156.90 ± 6.24	157.24 ± 6.07	156.07 ± 6.42	156.54 ± 6.55	<0.001
Weight (kg)	59.08 ± 10.93	57.14 ± 10.07	61.86 ± 10.28	62.86 ± 12.61	<0.001
Waist (cm)	78.94 ± 10.56	76.45 ± 9.68	82.4 ± 10.32	83.96 ± 10.93	<0.001
SBP (mmHg)	120.95 ± 16.52	116.64 ± 13.60	126.8 ± 17.06	129.75 ± 19.46	<0.001
DBP (mmHg)	74.04 ± 10.74	71.6 ± 9.24	77.22 ± 10.95	79.12 ± 12.51	<0.001
TG (mmol/L)	1.32 ± 1.06	1.09 ± 0.76	1.56 ± 1.24	1.88 ± 1.43	<0.001
TC (mmol/L)	4.41 ± 1.04	4.27 ± 0.83	4.58 ± 1.30	4.73 ± 1.26	<0.001
LDL (mmol/L)	2.68 ± 0.86	2.53 ± 0.73	2.87 ± 1.01	3.01 ± 1.01	<0.001
HDL (mmol/L)	1.27 ± 0.33	1.31 ± 0.30	1.21 ± 0.39	1.17 ± 0.35	<0.001
UA (µmol/L)	268.49 ± 73.87	261.21 ± 66.58	277.23 ± 77.84	284.40 ± 88.39	<0.001
FG (mmol/L)	5.98 ± 2.36	4.86 ± 0.51	6.43 ± 0.24	9.24 ± 3.65	<0.001
HbA1c (%)	5.16 ± 1.29	4.72 ± 0.48	5.00 ± 0.69	6.78 ± 2.08	<0.001

Abbreviation: SBP, systolic blood pressure; DBP, diastolic blood pressure; TG, triglycerides; TC, total cholesterol; LDL, low-density lipoprotein cholesterol; HDL, high density lipoprotein cholesterol; UA, uric acid; FG, fasting glucose; HbA1c, hemoglobin A1c.

**Table 3:** Associations of the plasma magnesium concentration with glucose parameters.

Characteristics	Plasma Mg (mmol/L)					*p*-Trend	Per 0.041 mmol/L of Mg	*p* Value
	<0.75	0.75– <0.85	0.85– <0.95	0.95– <1.05	≥1.05			
NFG/T2DM	OR (95%CI)			OR (95%CI)			OR (95%CI)	
Model 1	3.99 (2.93–5.42)	1.55 (1.25–1.92)	Reference	0.88 (0.63–1.25)	1.03 (0.48–2.22)	<0.001	0.81 (0.77–0.84)	<0.001
Model 2 ^#^	3.77 (2.71–5.25)	1.53 (1.22–1.93)	Reference	0.85 (0.59–1.22)	1.09 (0.48–2.48)	<0.001	0.82 (0.78–0.86)	<0.001
NFG/IFG	OR (95%CI)			OR (95%CI)			OR (95%CI)	
Model 1	4.05 (3.01–5.45)	1.49 (1.26–1.78)	Reference	0.94 (0.72–1.22)	0.69 (0.36–1.34)	<0.001	0.81 (0.78–0.85)	<0.001
Model 2 *	4.08 (2.92–5.71)	1.47 (1.21–1.79)	Reference	0.83 (0.62–1.12)	0.69 (0.32–1.45)	<0.001	0.81 (0.77–0.84)	<0.001
NFG/HbA1c-hyperglucose	OR (95%CI)			OR (95%CI)			OR (95%CI)	
Model 1	3.90 (2.70–5.63)	1.68 (1.27–2.22)	Reference	0.87 (0.55–1.39)	1.28 (0.49–3.30)	<0.001	0.80 (0.76–0.85)	<0.001
Model 2 ^&^	3.53 (2.39–5.19)	1.62 (1.21–2.16)	Reference	0.85 (0.52–1.38)	1.35 (0.50–3.62)	<0.001	0.82 (0.78–0.87)	<0.001

Model 2 ^#^: Adjusted for district, age group, Tg, CRP and Ca; Model 2 *: Adjusted for district, age group, Tg, CRP, BMI, Ca and rs3740393; Model 2 ^&^: Adjusted for district, age group, Ca, SBP and CRP.

**Table 4:** Daily dietary nutrient intake of the subjects.

Nutrients	Total (*n* = 2469)	NFG (*n* = 1533)	IFG (*n* = 462)	T2DM (*n* = 451)	*p* Value
Energy (kcal)	1964.71 ± 749.90	1944.88 ± 757.26	1989.52 ± 727.68	2006.67 ± 746.36	0.224
Protein (g)	58.77 ± 24.00	58.49 ± 24.33	59.37 ± 22.61	59.12 ± 24.29	0.742
Fat (g)	25.78 ± 21.12	25.92 ± 21.54	26.22 ± 21.23	24.85 ± 19.51	0.567
Carbohydrate (g)	408.78 ± 175.82	406.15 ± 177.04	408.81 ± 170.66	417.71 ± 176.94	0.471
Calcium (mg)	366.04 ± 290.27	372.95 ± 293.96	359.96 ± 290.34	348.76 ± 277.02	0.263
Magnesium (mg)	409.12 ± 176.50	410.97 ± 179.93	401.09 ± 167.19	411.03 ± 174.14	0.555

**Table 5:** Associations of the dietary magnesium concentration with glucose parameters.

Sextile of Dietary Magnesium Intake (mg/d)							
Model	S1	S2	S3	S4	S5	S6	*p*
	<321.35	321.35–<365.62	365.62–<401.16	401.16–<439.47	439.47–<492.89	>492.89	
NFG/IFG		OR (95%CI)					
Model 1	1	0.788 (0.595, 1.043)	0.810 (0.612, 1.071)	0.761 (0.575, 1.009)	0.594 (0.446, 0.791)	0.657 (0.495, 0.873)	0.009
Model 2	1	0.754 (0.552, 1.029)	0.742 (0.541, 1.018)	0.801 (0.582, 1.102)	0.541 (0.392, 0.748)	0.653 (0.472, 0.902)	0.009
Model 3	1	0.736 (0.527, 1.028)	0.730 (0.520, 1.024)	0.797 (0.566, 1.121)	0.535 (0.378, 0.755)	0.611 (0.431, 0.866)	0.011
NFG/T2DM		OR (95%CI)					
Model 1	1	0.883 (0.619, 1.261)	0.879 (0.615, 1.257)	0.877 (0.614, 1.251)	0.721 (0.502, 1.035)	0.745 (0.519, 1.070)	0.501
Model 2	1	0.862 (0.586, 1.269)	0.830 (0.560, 1.229)	0.964 (0.650, 1.432)	0.680 (0.457, 1.013)	0.781 (0.523, 1.166)	0.428
Model 3	1	0.787 (0.514, 1.205)	0.718 (0.465, 1.108)	0.873 (0.564, 1.350)	0.625 (0.404, 0.967)	0.707 (0.456, 1.098)	0.342

Model 1: unadjusted; Model 2 added age, education, district, residences; Model 3: added SBP, DBP, BMI, waist, TG, TC, HDL, LDL, UA.

**Table 6:** Consistency analysis of the dose–response effect of dietary magnesium and plasma magnesium on glucose parameters.

Tertiles of Dietary Magnesium Intake (mg/Day)				
Model	T1	T2	T3	*p*
	<330 mg/day	330–<408 mg/day	≥408 mg/d	
Plasma Mg(mmol/L)	0.86 ± 0.09	0.86 ± 0.08	0.87 ± 0.09	0.139
NFG/FG		OR (95%CI)		
Model 1	1	0.682 (0.652, 1.031)	0.671 (0.539, 0.836)	0.001
Model 2	1	0.795 (0.614, 1.030)	0.683 (0.530, 0.881)	0.013
Model 3	1	0.766 (0.578, 1.014)	0.632 (0.478, 0.837)	0.006
NFG/T2DM		OR (95%CI)		
Model 1	1	0.808 (0.605, 1.081)	0.735 (0.558, 0.967)	0.090
Model 2	1	0.785 (0.571, 1.081)	0.764 (0.560, 1.043)	0.212
Model 3	1	0.720 (0.504, 1.028)	0.697 (0.489, 0.993)	0.111

Model 1: unadjusted; Model 2: added age, education, district, residences; Model 3: added SBP, DBP, BMI, waist, TG, TC, HDL, LDL, UA.

## Data Availability

Data are contained within the article.
